# Supplementary material for: HIVprotI: an integrated web based platform for prediction and design of HIV proteins inhibitors
Source: J Cheminform. 2018 Mar 9;10:12. doi: 10.1186/s13321-018-0266-y (PMC5845081; doi:10.1186/s13321-018-0266-y)
Supplement: Supplementary file 2 — Additional file 2. Source code of HIVProtI web server. [file 13321_2018_266_MOESM2_ESM.zip › HIVprotI_Source-code/hivprotI/weka-3-6-6/doc/weka/gui/treevisualizer/TreeVisualizer.html]

TreeVisualizer


---


|  |  |  |
| --- | --- | --- |
| **Overview** \| **Package** \| **Class** \| **Tree** \| **Deprecated** \| **Index** \| **Help** \| **Weka's home** | |  |
| **PREV CLASS**   NEXT CLASS | **FRAMES**    **NO FRAMES**     **All Classes** |  |
| SUMMARY: NESTED \| FIELD \| CONSTR \| METHOD | DETAIL: FIELD \| CONSTR \| METHOD |  |


---


## weka.gui.treevisualizer Class TreeVisualizer

```
java.lang.Object
  java.awt.Component
      java.awt.Container
          javax.swing.JComponent
              javax.swing.JPanel
                  weka.gui.visualize.PrintablePanel
                      weka.gui.treevisualizer.TreeVisualizer
```

**All Implemented Interfaces:**: java.awt.event.ActionListener, java.awt.event.ItemListener, java.awt.event.MouseListener, java.awt.event.MouseMotionListener, java.awt.image.ImageObserver, java.awt.MenuContainer, java.io.Serializable, java.util.EventListener, javax.accessibility.Accessible, PrintableHandler

---

``` public class TreeVisualizer extends PrintablePanel implements java.awt.event.MouseMotionListener, java.awt.event.MouseListener, java.awt.event.ActionListener, java.awt.event.ItemListener ```

Class for displaying a Node structure in Swing.

To work this class simply create an instance of it.

Assign it to a window or other such object.

Resize it to the desired size.

When using the Displayer hold the left mouse button to drag the
tree around.

Click the left mouse button with ctrl to shrink the size of the tree
by half.

Click and drag with the left mouse button and shift to draw a box,
when the left mouse button is released the contents of the box
will be magnified
to fill the screen.

Click the right mouse button to bring up a menu.

Most options are self explanatory.

Select Auto Scale to set the tree to it's optimal display size.

**Version:**
:   $Revision: 7386 $

**Author:**
:   Malcolm Ware (mfw4@cs.waikato.ac.nz)

**See Also:**: Serialized Form

---

**Nested Class Summary**

**Nested classes/interfaces inherited from class javax.swing.JComponent**

`javax.swing.JComponent.AccessibleJComponent`

**Nested classes/interfaces inherited from class java.awt.Component**

`java.awt.Component.BaselineResizeBehavior`


| **Field Summary** | |
| --- | --- |
| `static java.lang.String` | `PROPERTIES_FILE`             the props file. |

**Fields inherited from class javax.swing.JComponent**

`TOOL_TIP_TEXT_KEY, UNDEFINED_CONDITION, WHEN_ANCESTOR_OF_FOCUSED_COMPONENT, WHEN_FOCUSED, WHEN_IN_FOCUSED_WINDOW`

**Fields inherited from class java.awt.Component**

`BOTTOM_ALIGNMENT, CENTER_ALIGNMENT, LEFT_ALIGNMENT, RIGHT_ALIGNMENT, TOP_ALIGNMENT`

**Fields inherited from interface java.awt.image.ImageObserver**

`ABORT, ALLBITS, ERROR, FRAMEBITS, HEIGHT, PROPERTIES, SOMEBITS, WIDTH`


**Constructor Summary**

`TreeVisualizer(TreeDisplayListener tdl,
Node n,
NodePlace p)`
  
          Constructs Displayer with the specified Node as the top
of the tree, and uses the NodePlacer to place the Nodes.

`TreeVisualizer(TreeDisplayListener tdl,
java.lang.String dot,
NodePlace p)`
  
          Constructs Displayer to display a tree provided in a dot format.


| **Method Summary** | |
| --- | --- |
| `void` | `actionPerformed(java.awt.event.ActionEvent e)`             Performs the action associated with the ActionEvent. |
| `void` | `fitToScreen()`             Fits the tree to the current screen size. |
| `void` | `itemStateChanged(java.awt.event.ItemEvent e)`             Performs the action associated with the ItemEvent. |
| `static void` | `main(java.lang.String[] args)`             Main method for testing this class. |
| `void` | `mouseClicked(java.awt.event.MouseEvent e)`             Does nothing. |
| `void` | `mouseDragged(java.awt.event.MouseEvent e)`             Performs intermediate updates to what the user wishes to do. |
| `void` | `mouseEntered(java.awt.event.MouseEvent e)`             Does nothing. |
| `void` | `mouseExited(java.awt.event.MouseEvent e)`             Does nothing. |
| `void` | `mouseMoved(java.awt.event.MouseEvent e)`             Does nothing. |
| `void` | `mousePressed(java.awt.event.MouseEvent e)`             Determines what action the user wants to perform. |
| `void` | `mouseReleased(java.awt.event.MouseEvent e)`             Performs the final stages of what the user wants to perform. |
| `void` | `paintComponent(java.awt.Graphics g)`             Updates the screen contents. |
| `void` | `setHighlight(java.lang.String id)`             Set the highlight for the node with the given id |

**Methods inherited from class weka.gui.visualize.PrintablePanel**

`getSaveDialogTitle, getWriter, getWriters, getXScale, getYScale, saveComponent, setSaveDialogTitle, setScale`

**Methods inherited from class javax.swing.JPanel**

`getAccessibleContext, getUI, getUIClassID, setUI, updateUI`

**Methods inherited from class javax.swing.JComponent**

`addAncestorListener, addNotify, addVetoableChangeListener, computeVisibleRect, contains, createToolTip, disable, enable, firePropertyChange, firePropertyChange, firePropertyChange, getActionForKeyStroke, getActionMap, getAlignmentX, getAlignmentY, getAncestorListeners, getAutoscrolls, getBaseline, getBaselineResizeBehavior, getBorder, getBounds, getClientProperty, getComponentPopupMenu, getConditionForKeyStroke, getDebugGraphicsOptions, getDefaultLocale, getFontMetrics, getGraphics, getHeight, getInheritsPopupMenu, getInputMap, getInputMap, getInputVerifier, getInsets, getInsets, getListeners, getLocation, getMaximumSize, getMinimumSize, getNextFocusableComponent, getPopupLocation, getPreferredSize, getRegisteredKeyStrokes, getRootPane, getSize, getToolTipLocation, getToolTipText, getToolTipText, getTopLevelAncestor, getTransferHandler, getVerifyInputWhenFocusTarget, getVetoableChangeListeners, getVisibleRect, getWidth, getX, getY, grabFocus, isDoubleBuffered, isLightweightComponent, isManagingFocus, isOpaque, isOptimizedDrawingEnabled, isPaintingForPrint, isPaintingTile, isRequestFocusEnabled, isValidateRoot, paint, paintImmediately, paintImmediately, print, printAll, putClientProperty, registerKeyboardAction, registerKeyboardAction, removeAncestorListener, removeNotify, removeVetoableChangeListener, repaint, repaint, requestDefaultFocus, requestFocus, requestFocus, requestFocusInWindow, resetKeyboardActions, reshape, revalidate, scrollRectToVisible, setActionMap, setAlignmentX, setAlignmentY, setAutoscrolls, setBackground, setBorder, setComponentPopupMenu, setDebugGraphicsOptions, setDefaultLocale, setDoubleBuffered, setEnabled, setFocusTraversalKeys, setFont, setForeground, setInheritsPopupMenu, setInputMap, setInputVerifier, setMaximumSize, setMinimumSize, setNextFocusableComponent, setOpaque, setPreferredSize, setRequestFocusEnabled, setToolTipText, setTransferHandler, setVerifyInputWhenFocusTarget, setVisible, unregisterKeyboardAction, update`

**Methods inherited from class java.awt.Container**

`add, add, add, add, add, addContainerListener, addPropertyChangeListener, addPropertyChangeListener, applyComponentOrientation, areFocusTraversalKeysSet, countComponents, deliverEvent, doLayout, findComponentAt, findComponentAt, getComponent, getComponentAt, getComponentAt, getComponentCount, getComponents, getComponentZOrder, getContainerListeners, getFocusTraversalKeys, getFocusTraversalPolicy, getLayout, getMousePosition, insets, invalidate, isAncestorOf, isFocusCycleRoot, isFocusCycleRoot, isFocusTraversalPolicyProvider, isFocusTraversalPolicySet, layout, list, list, locate, minimumSize, paintComponents, preferredSize, printComponents, remove, remove, removeAll, removeContainerListener, setComponentZOrder, setFocusCycleRoot, setFocusTraversalPolicy, setFocusTraversalPolicyProvider, setLayout, transferFocusBackward, transferFocusDownCycle, validate`

**Methods inherited from class java.awt.Component**

`action, add, addComponentListener, addFocusListener, addHierarchyBoundsListener, addHierarchyListener, addInputMethodListener, addKeyListener, addMouseListener, addMouseMotionListener, addMouseWheelListener, bounds, checkImage, checkImage, contains, createImage, createImage, createVolatileImage, createVolatileImage, dispatchEvent, enable, enableInputMethods, firePropertyChange, firePropertyChange, firePropertyChange, firePropertyChange, firePropertyChange, getBackground, getBounds, getColorModel, getComponentListeners, getComponentOrientation, getCursor, getDropTarget, getFocusCycleRootAncestor, getFocusListeners, getFocusTraversalKeysEnabled, getFont, getForeground, getGraphicsConfiguration, getHierarchyBoundsListeners, getHierarchyListeners, getIgnoreRepaint, getInputContext, getInputMethodListeners, getInputMethodRequests, getKeyListeners, getLocale, getLocation, getLocationOnScreen, getMouseListeners, getMouseMotionListeners, getMousePosition, getMouseWheelListeners, getName, getParent, getPeer, getPropertyChangeListeners, getPropertyChangeListeners, getSize, getToolkit, getTreeLock, gotFocus, handleEvent, hasFocus, hide, imageUpdate, inside, isBackgroundSet, isCursorSet, isDisplayable, isEnabled, isFocusable, isFocusOwner, isFocusTraversable, isFontSet, isForegroundSet, isLightweight, isMaximumSizeSet, isMinimumSizeSet, isPreferredSizeSet, isShowing, isValid, isVisible, keyDown, keyUp, list, list, list, location, lostFocus, mouseDown, mouseDrag, mouseEnter, mouseExit, mouseMove, mouseUp, move, nextFocus, paintAll, postEvent, prepareImage, prepareImage, remove, removeComponentListener, removeFocusListener, removeHierarchyBoundsListener, removeHierarchyListener, removeInputMethodListener, removeKeyListener, removeMouseListener, removeMouseMotionListener, removeMouseWheelListener, removePropertyChangeListener, removePropertyChangeListener, repaint, repaint, repaint, resize, resize, setBounds, setBounds, setComponentOrientation, setCursor, setDropTarget, setFocusable, setFocusTraversalKeysEnabled, setIgnoreRepaint, setLocale, setLocation, setLocation, setName, setSize, setSize, show, show, size, toString, transferFocus, transferFocusUpCycle`

**Methods inherited from class java.lang.Object**

`equals, getClass, hashCode, notify, notifyAll, wait, wait, wait`

**Field Detail**

### PROPERTIES\_FILE

```
public static final java.lang.String PROPERTIES_FILE
```

:   the props file.

    **See Also:**: Constant Field Values


**Constructor Detail**

### TreeVisualizer

```
public TreeVisualizer(TreeDisplayListener tdl,
                      java.lang.String dot,
                      NodePlace p)
```

:   Constructs Displayer to display a tree provided in a dot format.
    Uses the NodePlacer to place the Nodes.

    **Parameters:**: `tdl` - listener: `dot` - string containing the dot representation of the tree to display: `p` - the algorithm to be used to position the nodes.

---


### TreeVisualizer

```
public TreeVisualizer(TreeDisplayListener tdl,
                      Node n,
                      NodePlace p)
```

:   Constructs Displayer with the specified Node as the top
    of the tree, and uses the NodePlacer to place the Nodes.

    **Parameters:**: `tdl` - listener.: `n` - the top Node of the tree to be displayed.: `p` - the algorithm to be used to position the nodes.


**Method Detail**

### fitToScreen

```
public void fitToScreen()
```

:   Fits the tree to the current screen size. Call this after
    window has been created to get the entrire tree to be in view
    upon launch.

---


### actionPerformed

```
public void actionPerformed(java.awt.event.ActionEvent e)
```

:   Performs the action associated with the ActionEvent.

    :   **Specified by:**: `actionPerformed` in interface `java.awt.event.ActionListener`
    :   **Parameters:**: `e` - the action event.

---


### itemStateChanged

```
public void itemStateChanged(java.awt.event.ItemEvent e)
```

:   Performs the action associated with the ItemEvent.

    :   **Specified by:**: `itemStateChanged` in interface `java.awt.event.ItemListener`
    :   **Parameters:**: `e` - the item event.

---


### mouseClicked

```
public void mouseClicked(java.awt.event.MouseEvent e)
```

:   Does nothing.

    :   **Specified by:**: `mouseClicked` in interface `java.awt.event.MouseListener`
    :   **Parameters:**: `e` - the mouse event.

---


### mousePressed

```
public void mousePressed(java.awt.event.MouseEvent e)
```

:   Determines what action the user wants to perform.

    :   **Specified by:**: `mousePressed` in interface `java.awt.event.MouseListener`
    :   **Parameters:**: `e` - the mouse event.

---


### mouseReleased

```
public void mouseReleased(java.awt.event.MouseEvent e)
```

:   Performs the final stages of what the user wants to perform.

    :   **Specified by:**: `mouseReleased` in interface `java.awt.event.MouseListener`
    :   **Parameters:**: `e` - the mouse event.

---


### mouseDragged

```
public void mouseDragged(java.awt.event.MouseEvent e)
```

:   Performs intermediate updates to what the user wishes to do.

    :   **Specified by:**: `mouseDragged` in interface `java.awt.event.MouseMotionListener`
    :   **Parameters:**: `e` - the mouse event.

---


### mouseMoved

```
public void mouseMoved(java.awt.event.MouseEvent e)
```

:   Does nothing.

    :   **Specified by:**: `mouseMoved` in interface `java.awt.event.MouseMotionListener`
    :   **Parameters:**: `e` - the mouse event.

---


### mouseEntered

```
public void mouseEntered(java.awt.event.MouseEvent e)
```

:   Does nothing.

    :   **Specified by:**: `mouseEntered` in interface `java.awt.event.MouseListener`
    :   **Parameters:**: `e` - the mouse event.

---


### mouseExited

```
public void mouseExited(java.awt.event.MouseEvent e)
```

:   Does nothing.

    :   **Specified by:**: `mouseExited` in interface `java.awt.event.MouseListener`
    :   **Parameters:**: `e` - the mouse event.

---


### setHighlight

```
public void setHighlight(java.lang.String id)
```

:   Set the highlight for the node with the given id

    :   **Parameters:**: `id` - the id of the node to set the highlight for

---


### paintComponent

```
public void paintComponent(java.awt.Graphics g)
```

:   Updates the screen contents.

    :   **Overrides:**: `paintComponent` in class `javax.swing.JComponent`
    :   **Parameters:**: `g` - the drawing surface.

---


### main

```
public static void main(java.lang.String[] args)
```

:   Main method for testing this class.

    :   **Parameters:**: `args` - first argument should be the name of a file that contains a tree discription in dot format.


---


|  |  |  |
| --- | --- | --- |
| **Overview** \| **Package** \| **Class** \| **Tree** \| **Deprecated** \| **Index** \| **Help** \| **Weka's home** | |  |
| **PREV CLASS**   NEXT CLASS | **FRAMES**    **NO FRAMES**     **All Classes** |  |
| SUMMARY: NESTED \| FIELD \| CONSTR \| METHOD | DETAIL: FIELD \| CONSTR \| METHOD |  |


---
